# Potential binding modes of the gut bacterial metabolite, 5-hydroxyindole, to the intestinal L-type calcium channels and its impact on the microbiota in rats

**DOI:** 10.1080/19490976.2022.2154544

**Published:** 2022-12-13

**Authors:** Barbora Waclawiková, Paulo Cesar Telles de Souza, Markus Schwalbe, Constantinos G. Neochoritis, Warner Hoornenborg, Sieger A. Nelemans, Siewert J. Marrink, Sahar El Aidy

**Affiliations:** a Host-Microbe Metabolic Interactions, Groningen Biomolecular Sciences and Biotechnology Institute (GBB), University of Groningen, Groningen, The Netherlands; b Molecular Microbiology and Structural Biochemistry (MMSB - UMR 5086), CNRS & University of Lyon, Lyon, France; c Department of Chemistry, University of Crete, Heraklion, Greece; d Department of Behavioral Neurosciences, Cluster Neurobiology, Groningen Institute of for Evolutionary Life Sciences (GELIFES), University of Groningen, Groningen, The Netherlands; e Department of Molecular Neurobiology, Groningen Institute for Evolutionary Life Sciences (GELIFES), University of Groningen, Groningen, The Netherlands; f Molecular Dynamics, Groningen Biomolecular Sciences and Biotechnology Institute and Zernike Institute for Advanced Materials, University of Groningen, Groningen, The Netherlands

**Keywords:** Microbiota, molecular dynamics, motility, L-type calcium channels, indole derivatives

## Abstract

Intestinal microbiota and microbiota-derived metabolites play a key role in regulating the host physiology. Recently, we have identified a gut-bacterial metabolite, namely 5-hydroxyindole, as a potent stimulant of intestinal motility via its modulation of L-type voltage-gated calcium channels located on the intestinal smooth muscle cells. Dysregulation of L-type voltage-gated calcium channels is associated with various gastrointestinal motility disorders, including constipation, making L-type voltage-gated calcium channels an important target for drug development. Nonetheless, the majority of currently available drugs are associated with alteration of the gut microbiota. Using 16S rRNA sequencing this study shows that, when administered orally, 5-hydroxyindole has only marginal effects on the rat cecal microbiota. Molecular dynamics simulations propose potential-binding pockets of 5-hydroxyindole in the α1 subunit of the L-type voltage-gated calcium channels and when its stimulatory effect on the rat colonic contractility was compared to 16 different analogues, *ex-vivo*, 5-hydroxyindole stood as the most potent enhancer of the intestinal contractility. Overall, the present findings imply a potential role of microbiota-derived metabolites as candidate therapeutics for targeted treatment of slow intestinal motility-related disorders including constipation.

## Introduction

The gastrointestinal tract is home to trillions of microbes. The gut microbiota produces a wide range of small bioactive molecules derived from various substrates, including dietary precursors and medications.^1,2^ Such microbial conversion represents a significant regulatory mechanism by which gut microbes can alter intestinal host physiology, including gastrointestinal motility.^3–6^ Recently, we have identified 5-hydroxyindole, a product of gut microbial conversion of the dietary supplement and antidepressant 5-hydroxytryptophan, as a potent accelerator of the gastrointestinal motility via its activation of L-type voltage-gated calcium channels (LTCCs) located on the colonic smooth muscle cells.^7^ These findings proposed 5-hydroxyindole as a potential therapeutic for gastrointestinal slow motility disorders, since dysregulation of LTCCs is associated with slow intestinal dysmotility.^8–10^ Slow intestinal motility disorders, such as constipation, is a common, debilitating motility disorder affecting up to 27% of the population.^11^ Widespread treatment of constipation is an administration of laxatives, however these commonly used drugs have been associated with significant changes in the gut microbiota composition,^12^ which might have implications for the development of unwarranted side effects. Therefore, in view of the significant effects, a medication might have on the gut microbiota composition, it is pivotal to explore the drug–microbiota interactions that can ultimately influence the host health and clinical outcomes.^13^

LTCCs are voltage-gated ion channels that are activated upon changes in the membrane potential. These channels, which are widely distributed in the human smooth and skeletal muscles,^14^ function via mediating the Ca^2+^ entry that triggers multitudes of Ca^2+^-dependent cellular events, such as contraction and secretion.^15,16^ LTCCs, also identified as Ca_V_1 channels, consist of an ion conducting transmembrane α1 subunit that co-assemble with auxiliary subunits including the extracellular α2δ, the intracellular β, and the transmembrane γ.^15,17^ LTCCs are present in four isoforms Ca_V_1.1, Ca_V_1.2, Ca_V_1.3 and Ca_V_1.4, with Ca_V_1.2 and Ca_V_1.3 isoforms ubiquitously expressed in many mammalian cells, such as smooth cells of gastrointestinal tract (Ca_V_1.2).^14^

Here, we further investigate the potential of the gut motility stimulator, 5-hydroxyindole,^7^ as a targeted treatment for gastrointestinal slow motility disorders. We explore the effect of 5-hydroxyindole on the rat cecal microbiota using 16S rRNA sequencing. Moreover, we study the binding site of 5-hydroxyindole on the LTCCs, combining experimental screening assays with molecular dynamics (MD) simulations. Finally, we determine the pharmacophore groups important for the 5-hydroxyindole activity of the intestinal contractility.

## Results

### 5-hydroxyindole has a marginal effect on the richness and composition of the cecal microbiota in wild-type Groningen rats

**Figure 1.**
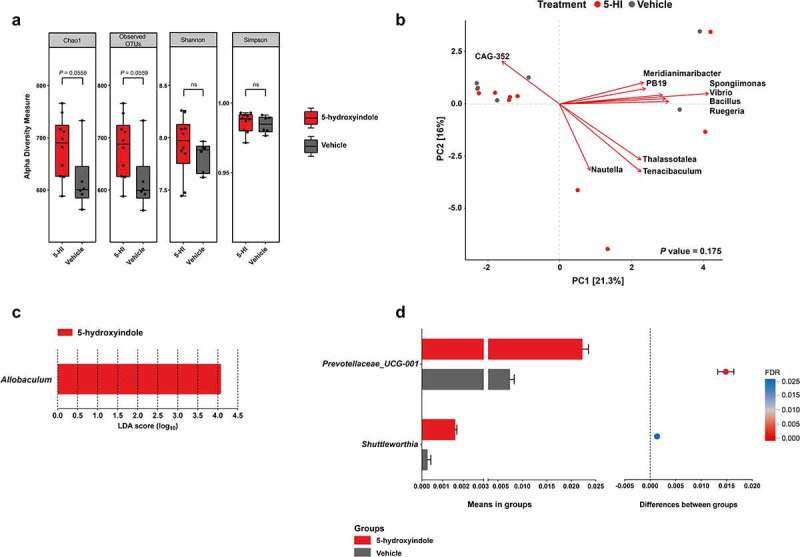
**5-hydroxyindole has a marginal effect on the cecal microbiota in wild-type Groningen rats**. (a) Comparison of cecal microbiota alpha diversity between 5-hydroxyindole-treated group (red bars) and vehicle-treated group (gray bars), including species richness (represented by Chao1 and Observed OTUs) and diversity (represented by Shannon and Simpson index). Data were analyzed using the Mann Whitney test (*P* value is indicated above the box plots; ns = not significant). Error bars represent SEM. The alpha diversity data can be found in Table A in S1 Table. (b) Principal component analysis (PCA) indicates no separation of 5-hydroxyindole-treated and vehicle-treated groups. Top 10 most contributing species are shown in the figure. The rest of the supporting data for the PCA analysis can be found in Table E and F in S1 Table. (c) LEfSe (Linear discriminant analysis Effect Size) for the 5-hydroxyindole-treated group. The length of the bar represents the log_10_ transformed Linear discriminant analysis (LDA) score for genera significantly changed in the 5-hydroxyindole-treated group, indicated by vertical dotted lines. (d) Difference in the abundance of the *Prevotellaceae_UCG-001* and *Shuttleworthia* genera in the 5-hydroxyindole-treated (red bars) and vehicle-treated (gray bars) groups. Left panel represents means in groups. Right panel represents differences between groups, where each dot is colored by its FDR value < .05. Significance was assessed by multiple comparison correction. Error bars represent SEM.

Recently, we showed that a daily oral administration of the gut microbiota-produced 5-hydroxyindole (30 mg/kg) to wild-type Groningen (WTG) rats for 11-day results in a significant decrease of the total gut transit time (TGTT).^7^ The dose for 5-hydroxyindole (30 mg/kg) was chosen based on a previous report.^18^ To investigate any possible effect of 5-hydroxyindole and the subsequent change in the gut motility on the microbiota composition, 16 cecal samples were collected (5-hydroxyindole-treated group (n = 10); vehicle-treated group (n = 6)) after the TGTT was measured and amplicon sequencing of the V3-V4 regions of the bacterial 16S gene was performed. Microbial richness, assessed by the Chao1 index and observed number of OTUs, showed a marginal but not significant (*P* value = .056) increase in 5-hydroxyindole-treated rats compared to the control group (Figure 1a; Table A in S1 Table). Next, the microbiota diversity was determined by Shannon’s H and Simpson’s index, both indices are used to measure similar parameters of alpha diversity.^19,20^ The diversity index did not differ between the treated and untreated groups (Figure 1a; Table A in S1 Table). The data highlight that 5-hydroxyindole has a negligible effect on the richness and no effect on the diversity of the cecal microbiota.

As a general exploratory analysis, principal component analysis (PCA) was performed, explaining 21.3% and 16% of the variance, respectively, and showed no significant difference between the 5-hydroxyindole and vehicle-treated groups (PERMANOVA: *P* value = .175, stratified *P* value = 1; Figure 1b; Table B in S1 Table). Next, LEfSe (Linear discriminant analysis Effect Size;^21^) was employed to complement our differential abundance analysis. The main discriminant feature separating the groups (5-hydroxyindole and vehicle groups) in WTG rats was the *Allobaculum* genus (Figure 1c). To support this analysis and investigate whether we can identify individual bacterial taxa to be affected by the 5-hydroxyindole treatment, pairwise comparisons of bacterial abundances were performed between 5-hydroxyindole-treated and vehicle-treated groups. Focusing on the phylum level, no significant changes were observed. On the family level, 5-hydroxyindole treatment seemed to only increase the abundance of family Yersiniaceae (*P* value = .03; **Table C in S1 Table**). On the genus level, 5-hydroxyindole treatment was associated with an increase in the abundance of *Allobaculum, Prevotellaceae_UCG-001, Serratia, Prevotellaceae_NK3B31_group, Shuttleworthia, Rikenellaceae_RC9_gut_group, Tuzzerella,Eubacterium_eligens_group, Parvibacter, Lachnospiraceae*_*NK4B4_group*), while reduced the abundance of *Acetatifactor* (*P* value < .05, unpaired *t* test with Welch’s correction) (Figure 1d; Table 1). Nonetheless, after multiple comparison corrections (false discovery rate; FDR), only *Prevotellaceae_UCG-001* and *Shuttleworthia* (FDR < 0.05) showed a significant increase in their abundance.

**Table 1.** Bacterial taxa on the genus level that were affected by the 5-hydroxyindole treatment. Significance was assessed by multiple comparison correction (FDR < .05). Marginal effect was assessed by unpaired t-test (P value < .05).

Genus	Avg(5-HI)	Sd(5-HI)	Avg(Veh)	Sd(Veh)	p.value	q.value	Difference
*Prevotellaceae_UCG-001*	0.0222	0.0040	0.0074	0.0023	<0.0001	<0.0001	0.0148
*Shuttleworthia*	0.0016	0.0003	0.0003	0.0004	0.0002	0.0174	0.0013
*Tuzzerella*	0.0012	0.0003	0.0006	0.0003	0.0014	0.0850	0.0007
*Allobaculum*	0.0309	0.0238	0.0076	0.0030	0.0128	0.4308	0.0234
*Parvibacter*	0.0002	0.0002	<0.0001	<0.0001	0.0136	0.4308	0.0002
*Prevotellaceae_NK3B31_*group	0.0027	0.0010	0.0011	0.0012	0.0205	0.4332	0.0017
*Rikenellaceae_RC9_*gut*_*group	0.0026	0.0012	0.0015	0.0005	0.0197	0.4332	0.0012
*Lachnospiraceae_NK4B4_*group	0.0001	0.0002	<0.0001	<0.0001	0.0283	0.4889	0.0001
*Serratia*	0.0108	0.0071	0.0033	0.0054	0.0326	0.5174	0.0075
*Eubacterium_eligens_*group	0.0008	0.0005	0.0004	0.0002	0.0419	0.6133	0.0004
*Acetatifactor*	0.0004	0.0003	0.0010	0.0004	0.0163	0.4332	−0.0006

Abbreviations: 5-HI, 5-hydroxyindole-treated group; Veh, Vehicle-treated group; avg, average; sd, standard deviation

Because the gut transit time was significantly affected in the 5-hydroxyindole-treated group,^7^ the TGTT was tested for its association with the abundance of genera using Spearman correlations. The correlation analysis revealed nine genera to be associated with the TGTT covariate (Spearman, *P* value < .05); Figure 1d; Table 2; Table D in S1 Table). Eight genera correlated negatively (*Lachnospiraceae_UCG-006, Lachnospiraceae_NK4B4_group, Tuzzerella, Prevotellaceae_NK3B31_group, Eubacterium_ventriosum_group, Prevotellaceae_UCG-001, Barnesiella, Bacteroides*) and one genus positively (*Anaerovibrio*). However, none of these associations could be detected as significant after multiple comparison correction (FDR). Taken together, the data analysis infers a minimal impact of 5-hydroxyindole treatment on the composition of the microbiota in the cecal samples of rats. Moreover, the TGTT covariate, which was shown to be significantly enhanced by 5-hydroxyindole treatment possibly via activation of LTCCs,^7^ is not significantly associated with the rat cecal bacterial composition, except for the increased relative abundance of the two bacterial taxa that were previously negatively associated with constipation.^22^ This gives an advantage for this microbiota-produced metabolite over several other available medications against constipation, such as commonly used laxatives, which have been linked to the significant changes in the gut microbiota composition^12^ and which might lead to a development of unwarranted side effects. Therefore, in view of these current results, which support the potential use of 5-hydroxyindole in cases of slow intestinal motility, we sought to further identify the binding site of the microbial-produced metabolite on the LTCCs and to determine the pharmacophore groups important for the 5-hydroxyindole activity, to be able to better understand the exact mechanisms how 5-hydroxyindole exerts its effect and how it influences the control of the complex gastrointestinal motility.

**Table 2.** Spearman correlations of bacterial taxa on the genus level that were marginally affected by the 5-hydroxyindole treatment.

Genus	Feature	Correlation	p.value	q.value
*Lachnospiraceae_UCG-006*	TGTT	−0.6702	0.0045	0.7847
*Lachnospiraceae_NK4B4_*group	TGTT	−0.6696	0.0045	0.7847
*Prevotellaceae_NK3B31_*group	TGTT	−0.6415	0.0074	0.8313
*Tuzzerella*	TGTT	−0.5670	0.0220	0.8457
*Eubacterium_ventriosum_*group	TGTT	−0.5457	0.0288	0.8457
*Prevotellaceae_UCG-001*	TGTT	−0.5404	0.0307	0.8457
*Barnesiella*	TGTT	−0.5200	0.0389	0.8457
*Bacteroides*	TGTT	−0.5107	0.0432	0.8457
*Anaerovibrio*	TGTT	0.6292	0.0090	0.8450

### 5-hydroxyindole potentially binds to the α1 subunit of the L-type voltage-gated calcium channels

**Figure 2.**
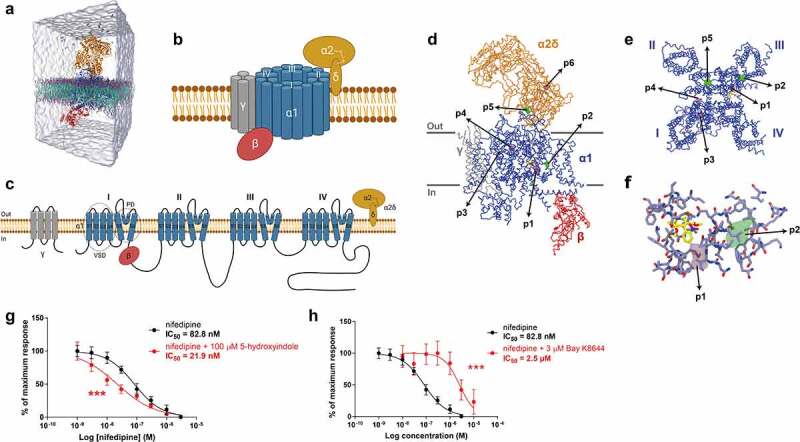
**5-hydroxyindole potentially binds to the α1 subunit of the L-type voltage-gated calcium channels**. (a) Representation of the simulation box used as initial configuration for the molecular dynamics simulations. The system contains CG models of L-type voltage-gated calcium channel Ca_V_1.1 complex embedded in a POPC bilayer. Solvent and ions are represented explicitly with 30 copies of 5-hydroxyindole (one of the ligands used for the study) were randomly placed in the water solution. (b – c) Schematic representation of L-type voltage-gated calcium channels (LTCCs) (b) and diagram of LTCCs topology (c). Adapted from^14,15,17^ (Created with BioRender.com). VSD, voltage sensing domain; PD pore domain; I–IV, four domains of α1 subunit; S1-S6, segments 1–6; P1-P2, supporting helices P1 and P2. (d – f) 5-hydroxyindole density obtained in the CG MD simulations projected on top of the cryo-EM structure of the L-type voltage-gated calcium channel Cav1.1 complex bound to nifedipine (pdb 6JP5). An overview of the whole structure of the complex, showing the backbone of the different subunits, with the pore-forming subunit α1 in blue and auxiliary subunits α2δ, β, and γ in Orange, red, and gray, respectively. Nifedipine is shown in yellow. Isosurfaces corresponding to a 1,000-fold higher than the environment are also displayed. Densities obtained from simulations starting from 6JP5 are shown in green while the data obtained with 3JBR is displayed in pink (d). Close look of the pockets in the pore-forming subunit α1 (e). Main residues of subunit α1 that are around nifedipine and of pockets p1 and p2 of 5-hydroxyindole (f). (g – h) Comparison of concentration-response curves of nifedipine alone (black) and 100 µM 5-HI + nifedipine (red) (g) and concentration-response curves of nifedipine alone (black) and 3 µM Bay K 8644 (red) (h). The response was normalized to maximum response and expressed as percentage. Concentration-response curves were fitted with nonlinear fit (Sigmoidal dose-response (variable slope)) and compared with Extra sum-of-squares F test (*** p < .001). Error bars represent SEM.

To study the binding mechanism of 5-hydroxyindole on the LTCCs, we explored all possible binding pockets of the LTCCs. We performed molecular dynamic (MD) simulations using the recent unbiased sampling approach^23^ based on the latest version of the coarse-grained (CG) Martini force-field.^24^ The CaV1.1 complex was embedded in a membrane model composed of 1-palmitoyl-2-oleoyl-sn-glycero-3-phosphatidylcholine (POPC) lipids, with a total of 30 copies of 5-hydroxyindole (equivalent to 11.1 mM concentration) randomly placed in the solvent (Figure 2a). Along the simulations, they can freely move in the box, exploring the possible binding pockets of 5-hydroxyindole in CaV1.1 complex. The Martini model has previously been used to accurately predict binding pockets and binding modes for a number of pharmaceutical relevant targets such as nuclear receptors, GPCRs, and kinases.^23^ Predictions of binding affinities are also possible, in cases with enough sampling.^23,25^ Thus, CG MD simulations could also provide useful insights for protein targets where the pockets are unknown as it is the case of LTCCs and 5-hydroxyindole. Two cryo-EM structures of the LTCC Ca_V_1.1 complex, both containing the pore-forming subunit α1 and auxiliary subunits α2δ, β, and γ (schematic representation of LTCCs is in Figure 2b–c), were used as references (3JBR (resolution 4.2 Å)^26^ and 6JP5 (resolution 2.9 Å))^15^ Of note, the Ca_V_1.1 isoform, present mostly in the skeletal muscle,^14^ is the only LTCC with available crystal structure,^15,26^ therefore the Ca_V_1.1 complex was used in our study instead of the Ca_V_1.2 complex that is present in the smooth muscle cells of the gastrointestinal tract.^14,27^ However, it was reported that the Ca_V_1.1 and Ca_V_1.2 contain highly conserved sequences.^15^

Our results from CG MD simulations indicated that 5-hydroxyindole may bind to only two sites of the Ca_V_1.1 complex: the pore-forming subunit α1 and auxiliary subunit α2δ (Figure 2d), which are the largest subunits of the Ca_V_1.1 complex. A total of 6 pockets were observed, all of them with occupancy of 3 orders of magnitude higher than the environment (membrane or water), which correspond roughly to a binding affinity of ~-15 to −20 kJ/mol. There were other pockets, but they showed lower affinity (≥ −10 kJ/mol). In the pore-forming subunit α1, there were 4 pockets, named p1-p4 (Figure 2d–e). Pocket p1 was located just below the binding site of nifedipine, an antagonist of LTCCs,^28^ between the S5_III_ and S6_III_ helices. Pocket p2 was also next to the nifedipine binding site, but not in direct contact with this ligand, next to the S5_III_ helix, on the opposite side of the nifedipine binding site. Pockets p3 and p4 were around 3 nm away from the nifedipine site and were located on opposite sides of the P1_I_ helix. Two additional pockets (p5 and p6) were observed in the auxiliary subunit α2δ. The pocket p5 was located at the interface between α1 and α2δ subunits. Finally, pocket p6 was in the Cache1 domain of the α2δ subunit.

Depending on the reference structure used in the CG MD simulations, p1 (pdb 3JBR, pink pocket in Figure 2f) or p2 (pdb 6JP5, green pocket in Figure 2f), are the main pockets near to the nifedipine site. Most known ligands of LTCCs, such as dihydropyridines (e.g. nifedipine and Bay K 8644), phenylalkylamines (e.g. verapamil) and benzothiazepines (e.g. diltiazem) bind to the α1 subunit.^17,28^ Therefore, we hypothesized that pockets p1 and p2 would most likely be the candidates involved in 5-hydroxyindole modulation of the activity of LTCCs.

To further confirm the MD simulation outcome, we employed an *ex vivo* organ bath system,^29^ where dissected proximal colonic tissues with intact mucosa from untreated wild-type Groningen rats were cut to approximately 3 mm rings and suspended in an organ bath as previously described.^7^ Nifedipine and 5-hydroxyindole were applied to the tissue mounted in the organ bath and colonic contractility was measured. A concentration-response curves for nifedipine (1 nM to 3 µM) in the 1) absence of 5-hydroxyindole and 2) the presence of 100 µM 5-hydroxyindole (added in the beginning to the tissue mounted in the organ bath to get maximum response of 5-hydroxyindole) were constructed. As a control, the LTCCs agonist, namely Bay K 8644, which has the same binding pocket as nifedipine,^15^ was used. In the presence of 100 µM 5-hydroxyindole, a leftward shift in the concentration-response curve for nifedipine was observed (Figure 2g). The IC_50_ value of nifedipine significantly decreased from 82.8 nM to 21.9 nM, suggesting that 5-hydroxyindole increases the binding affinity of LTCCs for nifedipine. In contrast, a rightward shift in the concentration-response curve for nifedipine was observed in the presence of Bay K 8644 (Figure 2h) and the IC_50_ value of nifedipine significantly increased from 82.8 nM to 2.5 µM, confirming that Bay K 8644 has the same binding pocket as nifedipine. Overall, the results indicate that 5-hydroxyindole affects the binding of nifedipine to LTCCs, but does not compete with it, reinforcing our hypothesis that 5-hydroxyindole can bind to either p1 or p2 binding pocket in the LTCCs.

### 5-hydroxyindoles analogues have various effects on rat colonic contractility

**Figure 3.**
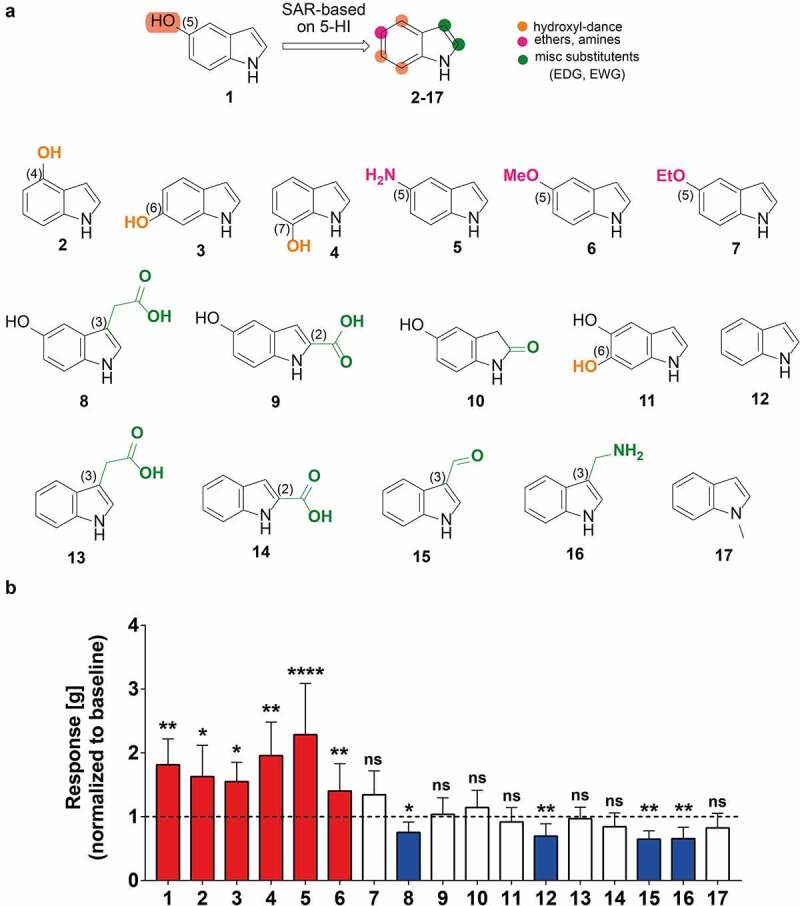
**5-hydroxyindoles analogues have various effects on rat colonic contractility**. (a) Chemical formulations of 5-hydroxyindole analogues. Top formula shows 5-hydroxyindole and structure-activity relationship based on 5-hydroxyindole. The hydroxyl-group dance on the indole core at the 4, 6, 7-position is highlighted by the Orange color. The 5-position is highlighted by the magenta color. The 2- and 3-position of the indole core which are prone to various electrophilic additions is highlighted by the green color. Formulas below show 5-hydroxyindole^1^ analogues: 4-hydroxyindole;^2^ 6-hydroxyindole;^3^ 7-hydroxyindole;^4^ 5-aminoindole;^5^ 5-methoxyindole;^6^ 5-ethoxyindole;^7^ 5-hydroxyindole-3-acetic acid;^8^ 5-hydroxyindole-2-carboxylic acid;^9^ 5-hydroxyoxindole;^10^ 5,6-dihydroxyindole;^11^ Indole;^12^ Indole-3-acetic acid;^13^ Indole-2-carboxylic acid;^14^ Indole-3-carboxaldehyde;^15^ (1 *H*-Indol-3-yl)methanamine;^16^ 1-methylindole.^17^ (b) Analogues of 5-hydroxyindole and their effect on the rat colonic contractility normalized to the respective baselines (n = 5–12; number of rat tissues used for each experiment). Red bars represent stimulants and blue bars represent inhibitors of the rat colonic contractility and white bars represent analogues with no effect. Data were analyzed using the Wilcoxon matched-pairs (before/after) signed rank test (*p < .05; **p < .01; **** p < .0001). Error bars represent SEM. Abbreviations: SAR, structure–activity relationship.

To determine the pharmacophore groups important for the enhancement of the rat colonic contractility, we performed an initial structure–activity relationship study by screening 16 indole analogues using the *ex-vivo* organ bath^29^ (Figure 3a). We compiled a pool of 17 easy-accessible indole derivatives, based on both the availability of the compounds and the main pharmacophore characteristics of the 5-hydroxyindole, e.g. the -OH, -NH along with the substitution pattern, specifically 5-hydroxyindole,^1^ 4-hydroxyindole,^2^ 6-hydroxyindole,^3^7-hydroxyindole,^4^ 5-aminoindole^5^ and 5-methoxyindole,^6^ 5-ethoxyindole,^7^ 5-hydroxyindole-3-acetic acid,^8^ 5-hydroxy-2-carboxylic acid,^9^ 5-hydroxyoxindole,^10^ 5,6-dihydroxyindole,^11^ indole,^12^ indole-3-acetic acid,^13^ indole-2-carboxylic acid,^14^ indole-3-carboxaldehyde,^15^(1*H*-Indol-3-yl) methanamine^16^ and 1-methylindole^17^ (Figure 3a). In addition, several of those analogues (i.e., compounds 1, 8, 10, 12, 13 and 15) are common metabolites in humans.^6,7,30,31^ The compounds (100 µM each) were applied to the rat colonic tissue mounted in the organ bath and left for 15 min to observe changes in the basal colonic contractility.

The starting point of our study was the hydroxyl group. Thus, we performed a hydroxyl-group dance on the indole core at the 4, 6, 7-position (Figure 3a; highlighted by the orange color). Then, we focused on the 5-position (Figure 3a; highlighted by the magenta color) by either removing the hydrogen bond possibility (introduction of ether groups) or switching the heteroatom to nitrogen (introduction of amines). Furthermore, we investigated the 2- and 3-position of the indole core which are prone to various electrophilic additions (Figure 3a; highlighted by the green color). Finally, we examined the importance of the free NH of our indole derivatives.

From the 17 screened compounds, hydroxyindoles and certain 5-substituted analogues, specifically 5-hydroxyindole,^1^ 4-hydroxyindole,^2^ 6-hydroxyindole,^3^ 7-hydroxyindole,^4^ 5-aminoindole^5^ and 5-methoxyindole^6^ were able to enhance the rat basal colonic contractility (Figure 3b). In contrast, four other compounds, 5-hydroxyindole-3-acetic acid,^8^ indole,^12^ indole-3-carboxaldehyde^15^ and (1*H*-Indol-3-yl)methanamine^16^ showed an inhibitory effect on the colonic contractility when compared to the respective baseline (Figure 3b). The rest of the tested compounds, 5-ethoxyindole,^7^ 5-hydroxy-2-carboxylic acid,^9^ 5-hydroxyoxindole,^10^ 5,6-dihydroxyindole,^11^ indole-3-acetic acid,^13^ indole-2-carboxylic acid^14^ and 1-methylindole,^17^ did not demonstrate any effect (Figure 3b).

This preliminary structure–activity relationship suggests that the oxygen heteroatom constitutes an important pharmacophore feature as both hydroxyl substituted indoles (at 4-,5-, 6- and 7-positions (compounds **1–4**)) and 5-methoxyindole^6^ stimulate the rat colonic contractility. The exchange of oxygen with nitrogen (e.g., the 5-aminoindole^5^) gave the same effect, suggesting the possibility of a hydrogen bonding on the 5-position as the most prominent feature. However, when we elongated the ether group at the 5-position (e.g., 5-ethoxyindole^7^), the corresponding compound could no longer stimulate the contractility, suggesting that more hydrophilic groups acting as hydrogen bonding donor are important. The additional hydrophobic/van der Waals interactions from the methoxy group can possibly hinder the binding of indole derivatives in this region of the LTCCs pocket. Substituted indole derivatives at the 2- and 3-positions bearing an -OH group (at the position 5) showed inhibitory (5-hydroxyindole-3-acetic acid^8^) or no effect (5-hydroxyoxindole,^10^ 5-hydroxyindole-2-carboxylic acid)^9^ At the same time, the absence of the hydroxyl group (compounds **12–17**) resulted in similar results. These results indicate a very specific substitution pattern which is influenced by the nature of the substituents, taking into consideration both their size and electronic factors; they might have rendered the molecules unsuitable for binding at the same pocket as 5-hydroxyindole^1^ and therefore they did not elicit the same effect. Noteworthy, the existence of an extra hydroxyl group on the 4-position (e.g., 5,6-dihydroxyindole^11^) and the NH substitution by methylation (e.g., 1-methylindole^17^) made the corresponding compounds inactive. Taken together, hydroxyindoles and certain analogues, namely 5-hydroxyindole,4-hydroxyindole,6-hydroxyindole, 7-hydroxyindole, 5-methoxyindole and 5-aminoindole were able to stimulate the rat colonic contractility, however the potency and the efficacy of these compounds remained unknown and was studied next.

### 5-hydroxyindole is the most potent stimulant of the rat colonic contractility among the other tested hydroxyindoles and their analogues

**Figure 4.**
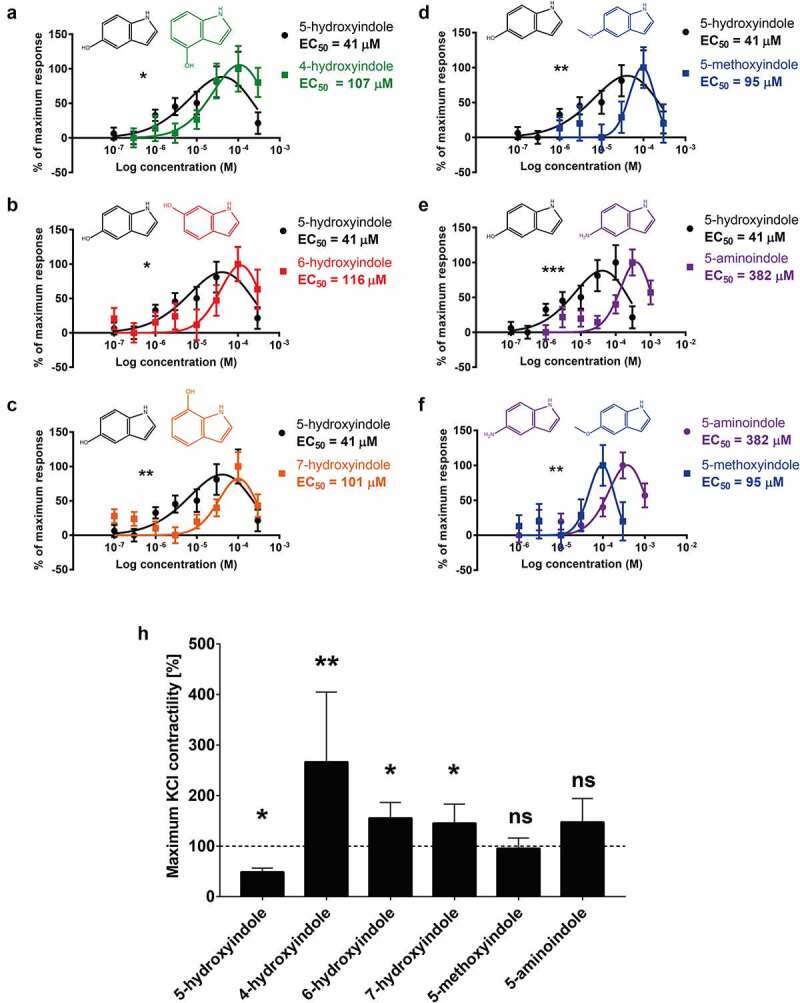
**5-hydroxyindole is the most potent stimulant of the rat colonic contractility among the other tested hydroxyindoles and their analogues**. (a – f) Comparisons of concentration-response curves (n = 6–9; number of rat tissues used for each experiment) of 5-hydroxyindole (black) and 4-hydroxyindole (green) (a), 5-hydroxyindole (black) and 6-hydroxyindole (red) (b), 5-hydroxyindole (black) and 7-hydroxyindole (Orange) (c), 5-hydroxyindole (black) and 5-methoxyindole (blue) (d), 5-hydroxyindole (black) and 5-aminoindole (purple) (e) and 5-aminoindole (purple) and 5-methoxyindole (blue) (f). In each panel, the response was normalized to maximum response and expressed as percentage. Each graph contains the chemical formulas of the studied molecules in the upper panel of each graph. Concentration-response curves were fitted with a nonlinear fit (Log(Gaussian)) and compared with Extra sum-of-squares F test (*p < .05; **p < .01; *** p < .001). Error bars represent SEM. (h) The effect of 5-hydroxyindole, 4-hydroxyindole, 6-hydroxyindole, 7-hydroxyindole, 5-methoxyindole and 5-aminoindole on the rat colonic contractility normalized to the maximum KCl-induced response (n = 6–7; number of rat tissues used for each experiment). Data were analyzed using the Wilcoxon matched-pairs (before/after) signed rank test (*p < .05; **p < .01; ns = not significant). Error bars represent SEM.

The stimulatory potential of 5-hydroxyindole, 4-hydroxyindole, 6-hydroxyindole, 7-hydroxyindole, 5-methoxyindole and 5-aminoindole was analyzed by construction of concentration-response curves. Increasing concentrations of these compounds (100 nM – 300 µM) were subsequently applied to the rat colonic tissue mounted in the organ bath and each concentration was incubated for 15 min. The results showed that 5-hydroxyindole has the lowest EC_50_ value of 41 µM, compared to EC_50_ values of 107, 116, 101, 95, and 382 µM depicted for 4-hydroxyindole, 6-hydroxyindole, 7-hydroxyindole, 5-methoxyindole, and 5-aminoindole, respectively (Figure 4a–e). Together, the data inferred that when the hydroxy group is replaced with methoxy or amino groups, the concentration needed to induce the stimulatory effect increased significantly. This was further confirmed by a comparison of the concentration-response curves of 5-hydroxyindole and 5-methoxyindole or 5-aminoindole (Figure 4d–e) or the concentration-response curves of 5-methoxyindole and 5-aminoindole (Figure 4f), where we observed significantly increased EC_50_ value for 5-methoxyindole and 5-aminoindole compared to the 5-hydroxyindole. Comparison of concentration-response curves of 5-hydroxyindole with 4-hydroxyindole, 6-hydroxyindole and 7-hydroxyindole showed significantly higher potency of 5-hydroxyindole (Figure 4a–c). In contrast, the concentration-response curves of 4-hydroxyindole, 6-hydroxyindole and 7-hydroxyindole did not show any significant differences (S1A – S1C Fig). The rank order of stimulatory potency of the compounds was estimated to be: 5-hydroxyindole > 5-methoxyindole > 4-hydroxyindole ≥ 6-hydroxyindole ≥ 7-hydroxyindole ≥ 5-aminoindole. Collectively, the half maximal effective concentration of the tested hydroxyindoles and their analogues reveal 5-hydroxyindole to be the most potent stimulant of the rat colonic contractility.

To evaluate the potential clinical relevance and safety of the tested hydroxyindoles and their analogues, the molecules were tested on KCl-induced response, since KCl is used to determine the maximum stimulatory effect that is considered to be safe within the physiological range.^32^ KCl (30 mM)^33^ was added to the tissue mounted in the organ bath to achieve the maximum KCl-induced response and subsequently 100 µM 5-hydroxyindole, 4-hydroxyindole, 6-hydroxyindole, 7-hydroxyindole, 5-methoxyindole or 5-aminoindole was added. 5-hydroxyindole significantly inhibited the KCl-induced response by 50%, whereas 4-hydroxyindole, 6-hydroxyindole and 7-hydroxyindole significantly increased the KCl-induced response by ~50% to ~150% (Figure 4h). The analogues of 5-hydroxyindole, 5-methoxyindole and 5-aminoindole did not show any significant change to KCl-induced response (Figure 4h). Taken together, these results infer that 5-hydroxyindole is the most clinically relevant compound since it does not exceed the maximum KCl-induced response.

### Comparison of 5-hydroxyindole possible binding sites in L-type voltage-gated calcium channels to other 5-hydroxyindole analogues

**Figure 5.**
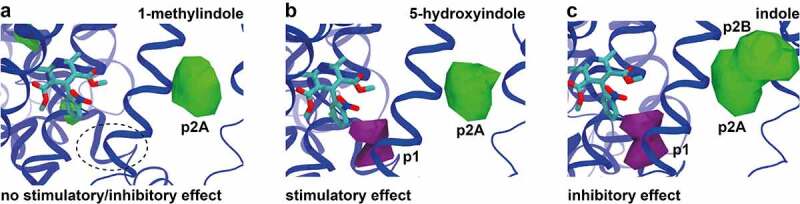
**Comparison of 5-hydroxyindole possible binding sites in L-type voltage-gated calcium channels to other 5-hydroxyindole analogues**. (a – c) Comparison of the pockets observed for all the ligands with no stimulatory/inhibitory effect (1-methylindole) (a), stimulatory effect (5-hydroxyindole) (b) and inhibitory effect (indole) (c) in subunit α1 that are around nifedipine (molecular structure in cyan color). Pockets p1 and p2 (p2A and p2B) are showed in purple and in green, respectively.

Given the possible involvement of pockets p1 (purple) and p2 (green) in the activity of 5-hydroxyindole (Figure 2f), additional MD simulations were performed, with the analysis focused on these two pockets. Two 5-hydroxyindole analogues with either no effect (1-methylindole) or inhibitory effect (indole) on the rat colonic contractility were used for comparison to 5-hydroxyindole. The non-stimulant, 1-methylindole, did not show binding to p1 (Figure 5a), while 5-hydroxyindole and indole both showed binding in this pocket near to nifedipine (Figure 5b–c). The comparison focusing on p2 showed a different picture. All the studied molecules seemed to bind to p2, but indole showed two possible binding modes: p2A, which is the same pocket observed for the other ligands; and p2B, which is only occupied by indole. Together, these results further explained the varying effect of the tested analogues and showed that binding to p2A would not promote relevant changes in the activity. On the other hand, p1 seemed to be the main pocket responsible for the stimulatory effect of the 5-hydroxyindole while p2B is related to the inhibitory response.

## Discussion

Together, our current study showed a marginal, presumably beneficial effect, of 5-hydroxyindole or its subsequent acceleration of the gut motility on the cecal microbiota composition when orally administered for 11 consecutive days to the WTG rats (Figure 1). We also demonstrate that 5-hydroxyindole potentially binds to the α1 subunit of LTCCs using CG MD simulations (Figure 2), where it possibly interferes with the binding of the main blocker of LTCCs, nifedipine (Figure 2g). Moreover, 5-hydroxyindole stood out as the most potent enhancer of the rat colonic contractility with the lowest EC_50_ value compared to the other hydroxyindoles and their analogues (Figure 4a–e), while its stimulatory effect remains within the physiological range, when compared to the maximum of KCl-induced response (Figure 4h).

Slow intestinal motility disorders, such as constipation, are highly prevalent gastrointestinal disorders in humans.^11^ We speculate that potential future application of 5-hydroxyindole as a targeted drug, may have a marginal impact on the host gut microbiota. This gives an advantage for this microbiota-produced metabolite over several other available medications against constipation, which have been linked to significant changes in the gut microbiota composition.^12^ Additionally, *Prevotella* genus has been reported to exhibit significant reduction in their relative abundance in the gut microbiome of the constipated patients compared to the controls.^22^ Thus, the observed significant increase in the abundance of *Prevotellaceae_UCG-001* upon the 5-hydroxyindole treatment may provide another beneficial effect for the consideration of 5-hydroxyindole as a treatment for constipated patients. Despite the considerable recent progress in describing the effects of the indole (tryptophan) metabolites on the composition and diversity of the intestinal microbiota,^34–37^ the overall impact of these metabolites on the host microbiota is contradicting and remains largely unknown. For example, Liang *et al*. showed that indole and its derivative, indole-3-acetic acid, significantly enhanced the richness, but not the diversity of the bacterial population in the cecal contents of piglets.^34^ On the other hand, other studies showed no effect of high levels of indole metabolites produced from tryptophan and Mediterranean diet on the alpha diversity in mice and humans, respectively.^36,37^

The CG MD simulations indicated that the binding pocket of 5-hydroxyindole is either located between S5_III_ and S6_III_ helices (p1 binding pocket) or next to S5_III_ helix (p2 binding pocket) (Figure 2f). These results need to be taken with caution as the accuracy of the Martini CG protein models depends on the resolution and conformational state of the reference structures used. On the other hand, the differences obtained with simulations may reflect the different conformations of the structures used: 3JBR is an apo-state structure (no ligand bound to the α1 subunit), while the 6JP5 structure was obtained with a ligand (nifedipine) bound to the α1 subunit. Further future studies need to be performed using all-atom MD simulations or combining Martini 3 and Gō models,^38,39^ allowing the transition of conformational states related to ligand binding. Notably, nifedipine and Bay K 8644 are both considered as allosteric modulators to the LTCCs.^28^ The closely related binding site of 5-hydroxyindole in LTCCs makes it plausible that 5-hydroxyindole can also act as an allosteric modulator to the LTCCs. Indeed, 5-hydroxyindole has been previously reported as an allosteric modulator of the 5-HT_3_ receptor^40^ and of alpha 7 nicotinic acetylcholine receptors (α7nAChRs).^41^

In our study, 5-hydroxyindole analogues showed different effects on rat colonic contractility (Figure 3). Within any given molecule or class of molecules, some functional groups are more important than others and alteration of functional groups can lead to, for example, enhanced activity.^42^ Also the location of a functional group can result different mode of actions.^42^ This is in an agreement with our data, where the rank order of stimulatory potency of the hydroxyindoles was estimated to be: 5-hydroxyindole > 5-methoxyindole > 4-hydroxyindole ≥ 6-hydroxyindole ≥ 7-hydroxyindole ≥ 5-aminoindole. In a previous study, it was also observed that hydroxyindoles can exhibit similar effects only with a varying potency.^43^ Moreover, 5-hydroxyindole significantly inhibited the KCl-induced response by 50%, whereas 4-hydroxyindole, 6-hydroxyindole and 7-hydroxyindole significantly increased the KCl-induced response by ~50% to ~150% (Figure 4h), while indole showed an inhibitory effect on the colonic contractility when compared to the respective baseline (Figure 3b). It is thus tempting to speculate that p2B is only occupied by indole and thus p2B is related to the inhibitory response (Figure 5c). On the other hand, p1 seemed to be the main pocket responsible for the stimulatory effect of the 5-hydroxyindole (Figure 5b). Potassium as a channel opener^44^ will most likely act as an allosteric modulator, binding to the same pocket or allosteric affecting the binding of 5-hydroxyindole Then, 5-hydroxyindole simultaneous binding to p1 and p2 might explain the reduction seen in Figure 4h.

Since LTCCs are associated with various gastrointestinal motility disorders, including constipation,^8–10^ they are an important target for drug development. LTCCs stimulants, such as Bay K 8644, have been reported to be unsuitable for clinical use.^14,17^ In contrast, 5-hydroxyindole is a naturally present compound, produced by the gut microbiota.^7^ This, together with having only marginal effects on the rat cecal microbiota composition (Figure 1) and being the most potent stimulator of rat colonic contractility among 16 different hydroxyindoles and their analogues (Figure 3–4), makes 5-hydroxyindole a potential candidate for targeted treatment of slow intestinal motility-related disorders including constipation. Future studies should focus on the confirmation of the proposed 5-hydroxyindole binding site in LTCCs as well as the determination of pharmacological properties of the compound in humans.

## Materials and methods

### Cecal samples collection

All animal procedures were approved by the Groningen University Committee of Animal experiments (approval number: AVD1050020197786) and were performed in adherence to the NIH Guide for the Care and Use of Laboratory Animals. Wild-type Groningen (WTG) male rats were orally administered either 30 mg/kg 5-hydroxyindole (H31859, Sigma) (n = 10) or vehicle (10% sucrose) (n = 6) for a period of 11 days and after measurement of TGTT (more detailed protocol can be found in^7^), the rats were anesthetized with isoflurane, killed and the whole cecum from every rat was collected and snap-frozen in a liquid nitrogen and stored in −80°C until further procedure.

### DNA isolation

DNA isolation followed a previously proposed protocol by.^45^ Briefly, about 500 mg of cecal material was resuspended in 750 µL of lysis buffer (500 mM NaCl, 50 mM Tris-HCl, 50 mM EDTA, 4% SDS) and transferred to a screw cap tube containing ~ 500 mg of 0.1 mm zirconium beads and four 3 mm glass beads. Samples were homogenized 3 × 1 min with 1-min intervals on ice in a mini bead-beater (Biospec, Bartlesville, USA). Subsequently, samples were further incubated at 95°C for 15 min and centrifuged at 16000 × g for 15 min at 4°C. All subsequent centrifugation steps were conducted under the same conditions. The supernatant was transferred to a clean tube and 20 µL of 10 M ammonium acetate was added and incubated on ice for 10 min before centrifugation. The supernatant was again transferred to a clean tube and one volume of 100% ice cold isopropanol was added. Samples were incubated on ice for 1.5–2 h and centrifuged. The supernatant was then aspirated and the pellet was washed with 200 µL of 70% ethanol with following centrifugation to collect the pellet. Ethanol was removed and tubes were left to air-dry for ~1 h before resuspension of the pellet in 200 µL of TE buffer.

### 16S rRNA sequencing

Illumina 16S rRNA gene amplicon libraries were generated and sequenced at Novogene (Bioinformatics Technology Co., Ltd., Beijing, China). In short, 16S rRNA genes of distinct regions (16SV3-V4) were amplified used specific primers (341 F and 806 R) with the barcode. All PCR reactions were carried out with Phusion High-Fidelity PCR Master Mix with GC Buffer (New England Biolabs). The PCR products were detected by 2% agarose gel electrophoresis and the samples were mixed equally according to the concentration of PCR products. After full mixing, the 2% agarose gel electrophoresis was used for the detection again and the target band was recovered by using the gel recovery kit provided by Qiagen company. The library was constructed by NEBNext Ultra™ IIDNA Library Prep Kit, and the constructed library was quantified by Qubit and Q-PCR. After the library was qualified, NovaSeq6000 was used for sequencing.

### Microbiota analysis

Paired-end reads were trimmed of their barcodes and sequencing primers and subsequently merged using FLASH (v1.2.11).^46^ Fastp was used for quality control and read filtering,^47^ while further VSEARCH was employed to detect chimera sequences by searching them against the Greengenes database.^48^ Filtered high-quality reads were then subjected to read denoising using DADA2 to obtain amplicon sequencing variants (ASVs), while making use of the QIIME2 software.^49,50^ Sequences with less than 5 counts were removed and the remaining ASVs were classified with Classify-sklearn in QIIME2 leveraging the Greengenes database.

For downstream analysis, QIIME2 was further used to assess alpha diversity. We used the *phyloseq* and *microbiome* packages in the statistical programming language *R* to process our data further.^51,52^ ASV absolute abundances were collapsed on genus and family level (**Table G** and **H in S1 Table**) and CSS normalized^53^ and the resulting abundance table (genus level) was used for principal component analysis (PCA) (supporting data for PCA analysis can be found in **Table E** and **F in S1 Table**). Significance of the model was determined by an ANOVA-like permutation test implemented in the *vegan* package^54^ (PERMANOVA results can be found in **Table B in S1 Table**). Differential abundance between control and 5-hydroxyindole-treated groups was assessed by unpaired *t* test with Welch’s correction. Significance was evaluated using FDR < 0.05. Marginal effect was assessed by *P* value < .05 (see main text for more details and Table 1). Additionally, LEfSe was used with default parameters to investigate differentially abundant taxa.^21^ Pairwise correlations between microbial taxa and TGTT were performed using the *associate* function of the *microbiome* package, using Spearman correlations. Significance was evaluated using FDR < 0.05. Marginal effect was assessed by *P* value < .05 (see main text for more details, Table 2**; Table D in S1 Table**).

### Coarse-grained models

All molecular dynamics (MD) simulations were performed using the Martini 3 Coarse-Grained (CG) force field.^24,55^ The CG protein model was generated using the new version of the program Martinize.^56,57^ Two cryo-EM structures of the L-type voltage-gated calcium channel Ca_V_1.1 complex, both containing the pore-forming subunit α1 and auxiliary subunits α2δ, β, and γ, were used as references: 3JBR (resolution 4.2 Å)^26^ and 6JP5 (resolution 2.9 Å).^15^ Given the dependence of Martini protein models on the reference structure, each cryo-EM structure was used to build an independent model. Such strategy allowed a better view of the possible pockets in the complex, as these cryo-EM structures were fairly different, giving the presence of a nifedipine ligand in one of the structures.^15^ Only the protein structures were considered in the model, the glycosylation, post-translational modifications, lipids, ligands, and calcium ions were removed from the structure. As a significant number of missing loops were also not included, the extra termini generated in the chains were capped to avoid influence of additional charged sites in the model. To further increase stability of the complex, elastic networks^58^ were applied to the whole complex, with a distance cutoff of 1.0 nm using a force constant of 1000 kJ mol^−1^ nm^−2^. CG models of the ligands were obtained according to the parametrization rules of Martini 3.^24,59^ Bond and angle parameters were parametrized using as reference an atomistic structure generated with the LigParGen server.^60^ Bond distances were optimized according to the center of geometry mapping (including the hydrogen atoms), which give a good compromise of molecular solvent-accessible surface area and bulk density.^24,59^ Three different ligands were considered, each one representing a class of effects: indole, which shows an inhibitory effect; 1-methyl indole, with no effect; and 5-hydroxyindole, with a stimulatory effect. In analogy with indole (which was already published with Martini 3), the model of 5-hydroxyindole and 1-methylindole were built using four beads connected with constraints, and one central bead, described as virtual site. In 5-hydroxyindole, the ethanol fragment was modeled by a SN6 bead while the aromatic amine group used a 2–1 mapping and TN6d bead. The remaining carbon-based aromatic fragments were represented by three TC5 beads with the central virtual bead receiving an extra label “e”. This label was added giving the possible influence of electron donating character of the hydroxyl group attached to the aromatic ring. For 1-methylindole, the N-methyl group was modeled by a SN1 bead with the carbon-based aromatic fragments modeled in the same way as used in indole and 5-hydroxyindole. Lipids models were based on the previous Martini 2 force-field,^55,61^ but now following the rules for mapping of Martini 3^24,59^ and also with adaptations in the bonded parameters inspired by the “extensible model” of Carpenter *et al*. 2018.^62^

### System setup and settings of the MD simulations

The Ca_V_1.1 complex was embedded in a membrane composed of 1-palmitoyl-2-oleoyl-sn-glycero-3-phosphatidylcholine (POPC) lipids. The simulation box with dimensions of 16 × 16 x 22 nm^3^ was built using the INSANE program.^61^ The pore-forming subunit α1 was positioned in the membrane according to the data derived from the OPM database.^63,64^ The orientation of the principal z-axis of this subunit was set to be parallel to the normal of the lipid bilayer. The system was solvated using water solution with 0.15 M concentration of NaCl, mimicking physiological conditions. A total of 30 copies of one of the ligands were randomly placed in the solvent, which is an equivalent to a water solution of around 11.1 mM. For each ligand, a separated box was built. The systems were equilibrated for 10 ns allowing protein side chains, membrane and the solvent to relax while the backbone was kept fixed using position restraints. The restraints were subsequently removed and the system was equilibrated for another 10 ns. The production simulations were performed for 30 µs. This procedure was repeated 10 times, resulting in a total sampling of 300 µs. Settings for the CG simulations followed the “new” Martini set of simulation parameters.^65^ Temperatures of the system were kept at 310 K with the velocity rescaling thermostat.^66^ For the pressure, we used the semi-isotropic coupling at 1 bar using the Parrinello-Rahman barostat.^67^ All simulations were performed with GROMACS (version 2020).^68^ A representation of the simulation box (after equilibration) is displayed in (Figure 2a).

### Analysis of the trajectories

Ligand density was used the determine the main pockets observed in the MD simulations. Firstly, the ligands were placed at the minimum distance to the complex in every snapshot of the trajectories with respect to the periodic boundary conditions. Then, the Ca_V_1.1 complex was positioned in the box center and its backbone was aligned with the GROMACS *gmx trjconv* tool^68^ using different subunits of the complex as reference. Preliminary analysis and visual inspection of the trajectories indicated that two subunits showed the most important pockets: pore-forming subunit α1 and auxiliary subunit α2δ. These two subunits were used as references for the alignments, generating two independent processed trajectories, which were independently analyzed. Given the relative movements between the different units of the complex, this procedure was used to precisely identify the position of the pockets in each subunit. The ligand density was obtained by computing the occupancy of the ligand in the three-dimensional space using the Volmap plugin of VMD (69). The grid points had a distance of 0.2 nm. All ligand bead sizes were taken into account. The radius of small beads is 0.23 nm; the one of tiny beads 0.19 nm. Binding free energies ($\Delta G_{bind}$) were estimated based on the populations of the ligand in the pockets (*p_pocket_*, mainly p1 and p2) and environments (*p_environment_*, considering water or membrane), according to $\Delta G_{bind}=-RTln\left(p_{pocket}/p_{enviroment}\right)$.

### Organ bath experiments

Wild-type Groningen (WTG) male rats were anesthetized with isoflurane, killed and a proximal colon was immediately removed and washed in 1X PBS and placed in 0.7% NaCl solution. Approximately 3 mm rings were cut and were placed in an organ bath (Tissue Bath Station with SSL63L force transducer, Biopac Systems Inc. Varna, Bulgaria) filled with Krebs–Henseleit solution (NaCl, 7.02 g/L; KCl, 0.44 g/L; CaCl_2_.2 H_2_O, 0.37 g/L; MgCl_2_.6H_2_O, 0.25 g/L; NaH_2_PO_4_.H_2_O 0.17 g/L; Glucose, 2.06 g/L; NaHCO_3_, 2.12 g/L) gassed with Carbogen gas mixture (5% CO_2_, balanced with O_2_) at 37°C. At the beginning of the experiment, tension of the intestine of 0.5–1 g was obtained by adjusting the stretcher. Under these conditions, colonic rings were equilibrated for at least 45–60 min with replacement of Krebs–Henseleit solution approximately every 15 min.

### Screening experiment

The screening of all the following compounds was performed by addition of 100 µM of each derivative to the tissue mounted in the organ bath to measure a specific contractile response of every derivative; 5-hydroxyindole (H31859), 4-hydroxyindole (219878), 7-hydroxyindole (CDS005198), 5-aminoindole hydrochloride (307203), 5-hydroxyindole-3-acetic acid (H8876), 5-hydroxyindole-2-carboxylic acid (418608), 5-hydroxyoxindole (CDS004194), 5,6-dihydroxyindole (CDS021567), Indole (W259306), Indole-3-acetic acid (I3750) and Indole-3-carboxaldehyde (129445) were purchased from Sigma-Aldrich Ltd. 6-hydroxyindole (023442), 5-ethoxyindole (078269) and (1 H-Indol-3-yl)methanamine (078831) were purchased from Fluorochem. 5-methoxyindole (M0731), Indole-2-carboxylic acid (I0332) and 1-methylindole (M0561) were purchased from TCI. MilliQ-filtered water was used to dissolve 5-hydroxyindole, 6-hydroxyindole and 5-aminoindole. DMSO was used to dissolve 4-hydroxyindole, 7-hydroxyindole, 5-methoxyindole, 5-ethoxyindole, 5,6-dihydroxyindole and indole. All other compounds were dissolved in ethanol. As control, 0.2% DMSO (solvent of: 4-hydroxyindole, 7-hydroxyindole, 5-ethoxyindole, 5,6-dihydroxyindole and indole), or 0.2% ethanol (solvent of 5-methoxyindole, 5-hydroxyindole-3-acetic acid, 5-hydroxyindole-2-carboxylic acid, 5-hydroxyoxindole, indole-3-acetic acid, indole-2-carboxylic acid, indole-3-carboxaldehyde, (1 H-Indol-3-yl)methanamine, 1-methylindole) was applied prior to addition of mentioned compounds to the tissue to check for any change in contractions. Each treatment lasted for ~15 min. Data was recorded BioPac Student Lab 4.1 (Build: Feb 12, 2015). Quantitative analysis of the organ bath recordings was performed as reported before.^7,70^ In short, each 15 min recording segment was selected in BioPac Student Lab 4.1 and FFT analysis was done with following settings: data were padded with zeros, mean was removed, and magnitude was displayed with linear transform, signal was processed using Hamming window. Afterward, the maximum amplitudes of the dominant frequencies obtained from FFT analysis were selected and analyzed in GraphPad Prism 7.

### Construction of concentration-response curves

Cumulative concentration-response curves were constructed by addition of increasing concentrations of studied drugs to the tissue mounted in the organ bath at intervals of 15 min (5 min for nifedipine). 5-hydroxyindole, 4-hydroxyindole, 6-hydroxyindole,7-hydroxyindole, 5-methoxyindole, 5-aminoindole were added in the concentrations ranging from 100 nm to 300 µM. Nifedipine was added in the concentrations ranging from 1 nM to 3 µM. Concentration-response curve for nifedipine in the presence of 100 µM 5-hydroxyindole or 3 µM Bay K 8644 were initiated, when maximum response of either 5-hydroxyindole or Bay K 8644 was achieved. As control, 0.2% DMSO was applied prior to addition of all compounds to the tissue to check for any change of contractions, except 5-hydroxyindole, 6-hydroxyindole and 5-aminoindole, which were dissolved in MilliQ-filtered water. Data was recorded BioPac Student Lab 4.1 (Build: Feb 12, 2015). Quantitative analysis of the organ bath recordings was performed as described above. Data were normalized in GraphPad Prism 7, where 0% and 100% was defined as the smallest and the largest mean, respectively, in each data set. Results were presented as percentage of a maximum response. The normalized data was fitted using the nonlinear regression. For nifedipine concentration-response curves, sigmoidal dose-response (variable slope) was used with the bottom and top constrained to 0 and 100, respectively. Every other concentration-response curve was fitted with nonlinear fit (Log(Gaussian)). Automatic outlier elimination option was selected with ROUT analysis (Q = 1%).

### KCl-induced response

Potassium chloride (KCl; 30 mM) was added to the tissue mounted in the organ bath to achieve the maximum KCl-induced response and contractions were recorded for 10 min. Subsequently, 100 µM 5-hydroxyindole, 4-hydroxyindole, 6-hydroxyindole, 7-hydroxyindole, 5-methoxyindole or 5-aminoindole was added and contractions were recorder for additional 15 min. Data was recorded BioPac Student Lab 4.1 (Build: Feb 12, 2015). Quantitative analysis of the organ bath recordings was performed as described above.

## Statistical analysis and nonlinear regression models

All statistical tests and nonlinear regression models were performed using GraphPad Prism 7. For alpha diversity, Mann Whitney test was used. For pairwise comparison (beta diversity) between groups, unpaired *t* test with Welch’s correction was used. Data are presented as mean ± SEM. Data were evaluated with FDR < 0.05 for significance and *P* value < .05 for marginal effect (see main text for more details). For correlations between cecal microbiota, 5-hydroxyindole treatment and TGTT, Spearman correlation was used. Data were evaluated with FDR < 0.05 for significance and *P* value < .05 for marginal effect (see main text for more details). For concentration-response curves, the nonlinear sigmoidal dose-response (variable slope) or log(Gaussian) regression was used. For comparison of concentration-response curves, extra sum-of-squares F test was used. For screening and KCl organ bath measurements, the Wilcoxon matched-pairs (before/after) signed rank test was used. Data are presented as mean ± SEM and p < .05 was considered statistically significant. The (n) refers to the number of rat tissues used for each experiment. Specific test, significance and (n) number are indicated in the Figure legends.

## Supplementary Material

Supplemental Material

## Data Availability

All sequencing data is available at PRJNA800624.
