# Supplementary figures and images for: Potential binding modes of the gut bacterial metabolite, 5-hydroxyindole, to the intestinal L-type calcium channels and its impact on the microbiota in rats
Source: Gut Microbes. 2022 Dec 13;15(1):2154544. doi: 10.1080/19490976.2022.2154544 (PMC9754111; doi:10.1080/19490976.2022.2154544)

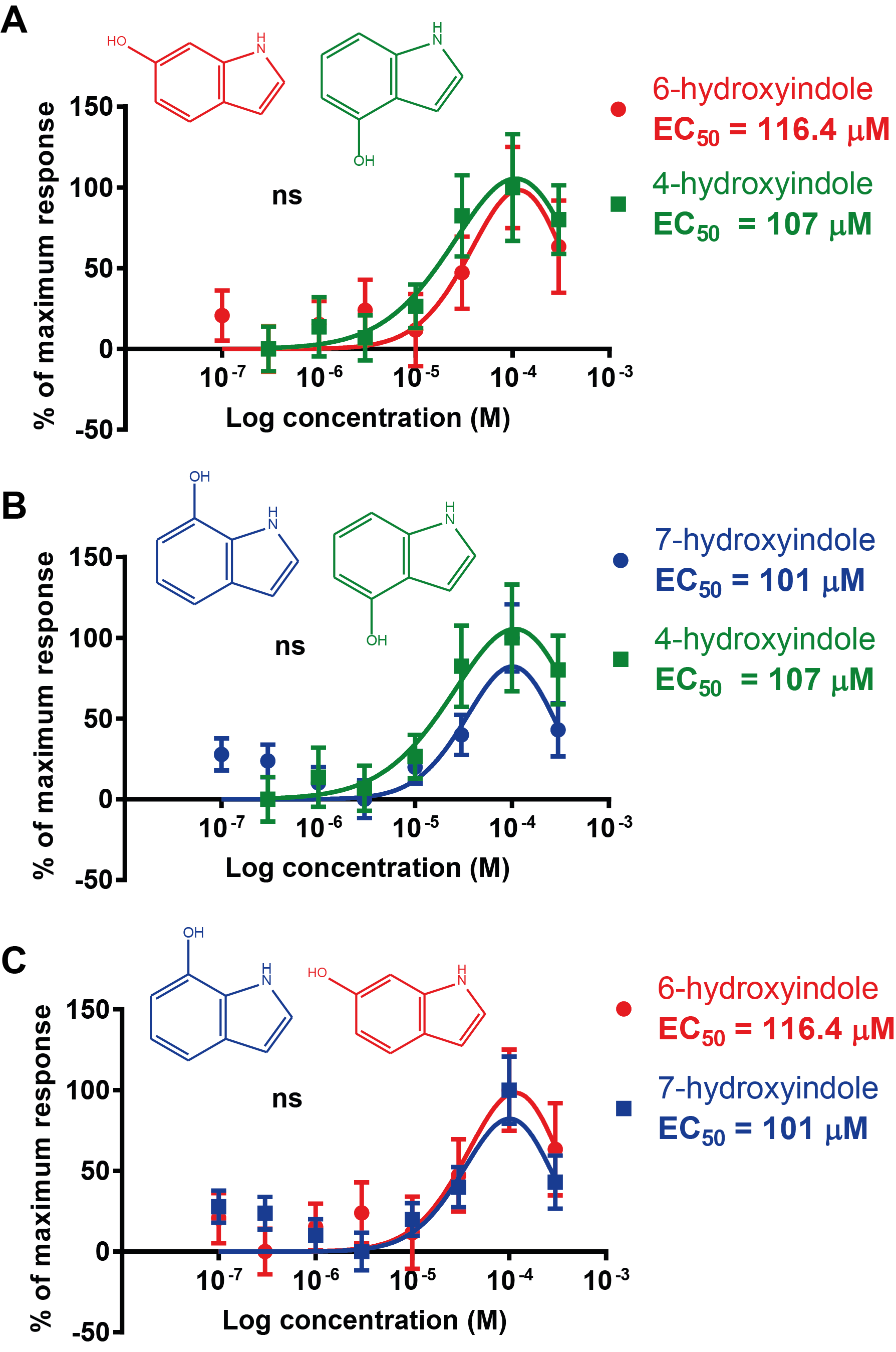

Supplement: Supplemental Material [file KGMI_A_2154544_SM8736.zip › S1 Fig.png]
